# *Borrelia spielmanii*–Associated Neuroborreliosis in Patient Receiving Rituximab, Belgium

**DOI:** 10.3201/eid3102.240777

**Published:** 2025-02

**Authors:** Timothée Carette, Louisien Lebrun, Benoît Kabamba-Mukadi, Jean-Marc Raymackers, Jean-Louis Bayart

**Affiliations:** Clinique Saint-Pierre Ottignies, Ottignies, Belgium (T. Carette, L. Lebrun, J.-M. Raymackers, J.L. Bayart); UCLouvain Institute of NeuroScience, Brussels, Belgium (L. Lebrun); UCLouvain Institut de Recherche Expérimentale et Clinique, Brussels (B. Kabamba-Mukadi); Cliniques Universitaires Saint-Luc, Brussels (B. Kabamba-Mukadi, J.-L. Bayart)

**Keywords:** bacteria, *Borrelia spielmanii*, neuroborreliose, neurolyme, rituximab, Belgium

## Abstract

A 58-year-old woman in Belgium with a history of follicular lymphoma treated with rituximab sought care for a rapid sensory-motor deficit. Seronegative neuroborreliosis caused by *Borrelia spielmanii* was diagnosed, likely related to humoral deficiency. High CXC motif chemokine ligand 13 levels and PCR confirmed the diagnosis. Ceftriaxone treatment led to full recovery.

Neuroborreliosis is an infectious disease of the nervous system caused by tickborne *Borrelia* species, primarily *Borrelia burgdorferi *sensu lato. Common species pathogenic to humans include *B. burgdorferi *sensu stricto, *B. afzelii*, and *B. garinii*. *Borrelia spielmanii*, identified as a distinct species in 2006, has been associated with Lyme disease but not with neuroborreliosis (*1*,*2*).

## The Study

A 58-year-old White woman in Belgium who had a history of follicular lymphoma treated with R-CHOP (rituximab monoclonal antibody, cyclophosphamide, hydroxydaunorubicin, vincristine, and prednisolone) and rituximab maintenance sought care for a 2-month history of worsening sensory-motor deficit in the distal right lower limb, accompanied by intense neuropathic pain and hyperesthesia. Electromyography of the right lower limb showed the absence of sural responses, severe axonal loss in both peroneal and tibial nerves, and absent F-waves, suggesting lumbar plexus involvement. Serum studies revealed an immunoglobulin profile altered by rituximab treatment (IgG 2.57 g/L [reference range (RR) 6.5–16 g/L], IgM 0.25 g/L [RR 0.50–3.00 g/L], IgA 0.51 g/L [RR 0.40–3.50 g/L]). We noted GM1 antibodies at low titers (IgG 1:80, IgM 1:32), whereas tests for other autoimmune and infectious markers (including for *Treponema pallidum* and herpes simplex virus 1 and 2) were negative. Results of serologic tests of cerebrospinal fluid (CSF) and serum samples for *Borrelia* were also negative (LIAISON *Borrelia* IgG and LIAISON *Borrelia* IgM II; DiaSorin, https://www.diasorin.com).

**Figure 1:**
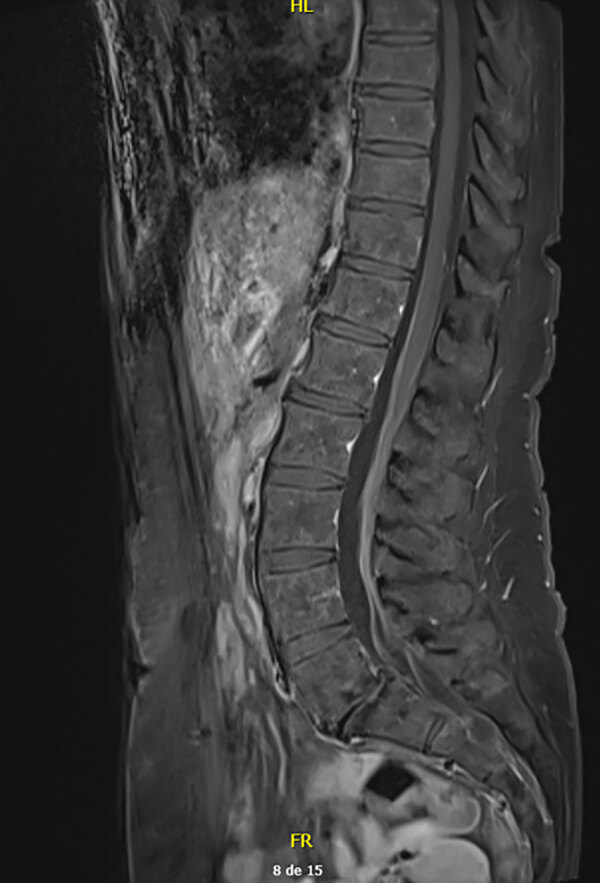
Contrast-enhanced T1-weighted magnetic resonance imaging scans of spine and cauda equina in study of *Borrelia spielmanii*–associated neuroborreliosis in patient receiving rituximab, Belgium. Subtle intradural contrast enhancement was noted after gadolinium injection. No hypertrophy of the cauda equina was found.

**Figure 2:**
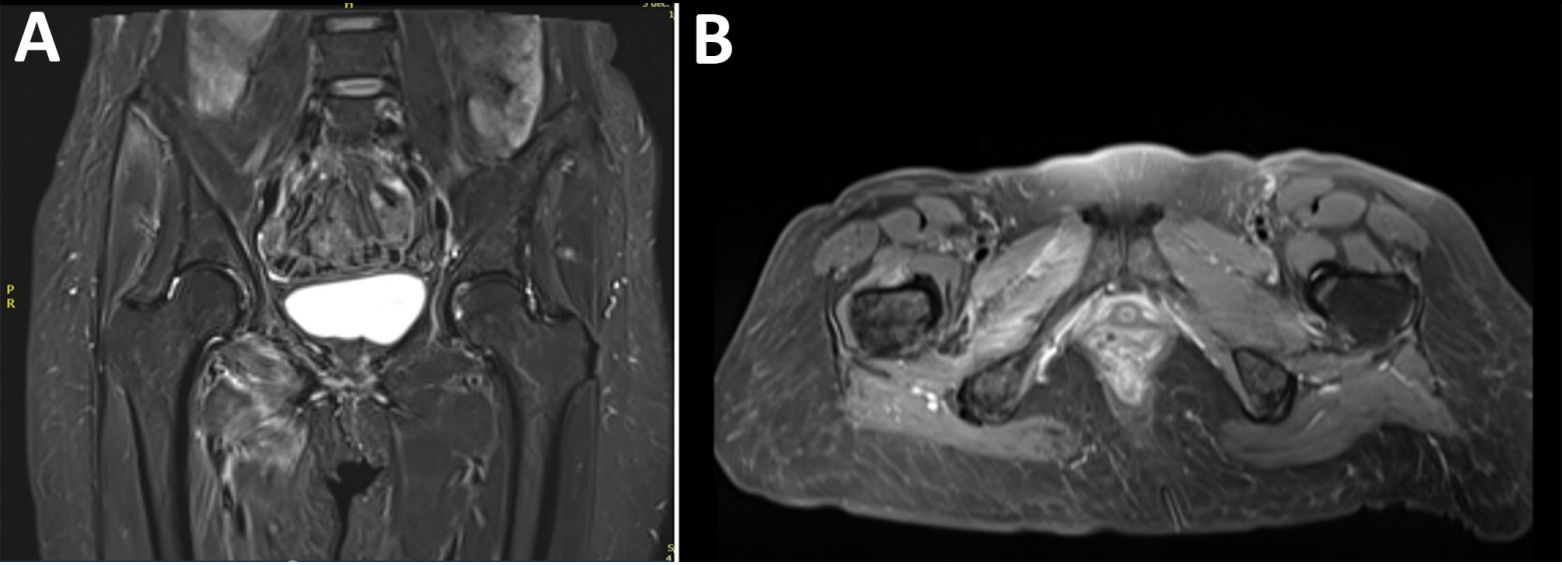
Coronal (A) and axial (B) T1 short-tau inversion recovery magnetic resonance imaging sequences of the hip in study of *Borrelia spielmanii*–associated neuroborreliosis in patient receiving rituximab, Belgium. Hyperintense infiltration of the right adductor muscle compartment (and partially of the hamstrings) was noted. No enhancement was seen after gadolinium injection.

Analysis of CSF revealed 33 leukocytes/mm^3^ (RR <5/mm^3^) with 30% neutrophils, 45% lymphocytes, and 25% macrophages and an elevated protein concentration of 84 mg/dL (RR 15–45 mg/dL). Isoelectric-focusing electrophoresis showed no specific CSF oligoclonal bands. Results of a multiplex PCR targeting 15 microorganisms (QIAGEN, https://www.qiagen.com) was negative, and no lymphomatous CSF infiltration was found by flow cytometry analysis. Results of brain magnetic resonance imaging (MRI) were unremarkable, whereas MRI of the spine showed subtle intradural contrast enhancement without hypertrophy of the cauda equina roots or enhancement of the lumbosacral plexus (Figure 1). MRI of the lumbar plexus showed hyperintense infiltration of the right adductor muscle compartment and partial infiltration of the hamstrings on short tau inversion recovery sequences, without gadolinium enhancement (Figure 2). Whole-body ^18^F-fluorodeoxyglucose positron emission tomography/computed tomography scans yielded negative results.

Given the patient’s rituximab-related hypogammaglobulinemia, we hypothesized that she did not develop antibodies to the causative infectious agent. Elevated CXC motif chemokine ligand (CXCL) 13 levels were found in CSF (>350 pg/mL). Considering the clinical manifestation and the high sensitivity and specificity of CXCL13 in acute neuroborreliosis (*3*), we initiated a 21-day course of ceftriaxone (2 g/d).

After antibiotic therapy, the patient showed near-complete recovery from paresis and substantial improvement in neuropathic pain. This response supported the diagnosis of *Borrelia*-induced radiculoplexitis. Subsequent results of PCR testing of CSF were positive for the *Borrelia* OspA gene; sequencing confirmed *B. spielmanii*. The patient then recalled a possible arthropod bite 3 months earlier and transient erythema in the right popliteal fossa weeks before onset of these symptoms. Further serologic testing at 8 weeks after symptom onset (4 weeks after treatment completion) remained negative. For this later sample, measurements with an alternative method (VIDAS Lyme IgG II and IgM II; bioMérieux, https://www.biomerieux.com) were also negative. As a complement, we performed Western blot testing for *Borrelia* (RecomLine *Borrelia* IgM and RecomLine *Borrelia* IgG; Mikrogen Diagnostik, https://www.mikrogen.de); results were negative.

## Conclusions

*B. spielmanii* is found mainly in central and western Europe and is relatively uncommon. In 2010, a study performed in Belgium identified *B. spielmanii* in only 1 of 524 *Ixodes* ticks analyzed (*4*). Radiculoneuritis is a frequent manifestation of neuroborreliosis. In this case, the patient’s history of transient erythema, rapid symptom evolution, and pleocytosis suggested an infectious etiology, after recurrent lymphomatous disease was ruled out. High intrathecal CXCL13 levels strongly indicated neuroborreliosis (*3*), a hypothesis confirmed by CSF PCR positivity for *B. spielmanii* despite negative results of serologic testing for *Borrelia*.

Current diagnostic criteria for definite neuroborreliosis by the European Federation of Neurologic Societies include neurologic symptoms suggestive of Lyme neuroborreliosis, CSF pleocytosis, and intrathecal *B. burgdorferi* antibody production (*5*). Our patient did not meet those criteria because of the absence of *B. burgdorferi*–specific antibodies, raising the question of a possible need to consider alternative or supportive criteria, such as *Borrelia burgdorferi *s.l. PCR positivity or high CSF CXCL13 levels.

*Borrelia burgdorferi *s.l. serology tests have imperfect sensitivity and specificity for Lyme disease; reports indicate 70%–97% sensitivity and 98%–99% specificity for neuroborreliosis (*6*). Given the rarity of *B. spielmanii*, the sensitivity and specificity of serologic testing for this species are unclear, requiring direct analysis methods such as PCR or CXCL13 measurement. The negative results obtained using different serologic methods and immunoblots highlight this report. Previous diagnosis of borreliosis might have been infructuous if the species involved was *B. spielmanii* missed with common serologic tests. In our evaluation, the LIAISON *Borrelia* IgG assay uses a recombinant VlsE antigen, which is an outer surface lipoprotein, whereas the LIAISON IgM II assay uses the outer surface protein OspC and VlsE antigen. Whether the antibodies used in these assays have different immunoreactivity between the various *B. burgdorferi *s.l. species needs further investigation. Indeed, Mechai et al. (*7*) recently highlighted that genetic diversity of *B. burgdorferi* and genetic distance from the antigen used in diagnostic kits can affect the serodiagnosis of Lyme disease when the test is based on the OspC antigen. Moreover, diagnostic performance varies greatly among the numerous assays on the market.

Assays using recombinant antigens are generally considered to have higher specificity but lower sensitivity than assays using whole-cell antigens (*8*). Of note, results for our patient using 2 distinct methods were negative, and the patient had completely negative immunoblots.

Rituximab, an anti-CD20 molecule, depletes B cells and impairs antibody production, possibly explaining the absence of seroconversion in our patient. In 2011, a study showed that rituximab prevented antibody production against *B. hermsii* in humanized mice (*9*). Four other cases of seronegative neuroborreliosis in rituximab-treated patients have been reported, diagnosed through CSF PCR or antibody positivity (*10*–*13*) (Appendix Table).

In patients with humoral immunity deficiency and symptoms suggestive of neuroborreliosis, tests for CXCL13 should be performed on CSF samples because high specificity and sensitivity, compared with PCR, makes this test a valuable diagnostic tool (*3*). CXCL13 levels decrease rapidly with treatment, serving as a marker of therapeutic response (*14*).

This report raises questions about the pathogenic potential of *B. spielmanii* in immunocompetent hosts. In 2019, a study compared patients with erythema migrans, including those on rituximab, and found higher signs of dissemination and *Borrelia* PCR positivity than in immunocompromised hosts (*15*).

In conclusion, this case of neuroborreliosis caused by *B. spielmanii* highlights the need for vigilance in rituximab-treated patients because their impaired antibody production can affect results of serologic testing. Further studies are needed to determine whether *B. spielmanii* can cause neuroborreliosis in immunocompetent patients. Alternative diagnostic methods, such as CXCL13 assays and *Borrelia*-specific PCR testing of CSF samples, should be considered, along with empirical therapy if clinical suspicion is high.

Appendix Additional information about *Borrelia spielmanii*–associated neuroborreliosis in patient receiving rituximab, Belgium.
