# Supplementary material for: Borrelia spielmanii–Associated Neuroborreliosis in Patient Receiving Rituximab, Belgium
Source: Emerg Infect Dis. 2025 Feb;31(2):341–4. doi: 10.3201/eid3102.240777 (PMC11845155; doi:10.3201/eid3102.240777)
Supplement: Appendix — Additional information about Borrelia spielmanii–associated neuroborreliosis in patient receiving rituximab, Belgium. [file 24-0777-Techapp-s1.pdf]

Article DOI: https://doi.org/10.3201/eid3102.240777

EID cannot ensure accessibility for supplementary materials supplied by authors. Readers who have difficulty accessing supplementary content should contact the authors for assistance.

Borrelia spielmanii–Associated Neuroborreliosis in Patient Receiving Rituximab, Belgium

Appendix

Appendix Table. Comparison between reported cases of neuroborreliosis in patients on Rituximab*

Study	Country/age/sex	Previous hemopathy	Previous treatment; date of last rituximab treatment	Symptoms	Additional tests	CSF data	*Borrelia* serology	CXCL13	*Borrelia* PCR	Treatment/ outcome
(*1*)	Germany/63 y/M	Chronic lymphocytic leukemia	Fludarabine/cyclophosphamide/ prednisone/rituximab; ongoing at onset	Neuropathic pain in the left upper arm and both upper legs, with hyperesthesia and slight facial paresis.	Brain MRI N/R; Spine MRI negative; EMG negative; whole-body CT scan negative; brain ^{18}F-FDG PET/CT negative	63 leukocyte/mm^3, lympho-monocytoid; 155 mg/dL proteins	Negative in blood, N/R in CSF	N/R	Positive in CSF for *B. garinii*	Ceftriaxone 2g 1x/d for 21 d/total recovery
(*2*)	Netherlands/66 y/F	Extranodal marginal zone B cell lymphoma stage IVB with extensive localizations (complete remission)	R-CHOP and maintenance with rituximab; ongoing at onset of borreliosis	Severe shooting pains in the back and both legs, followed by deafness, nausea, vertigo and headache. No paresis.	Brain/Spine MRI negative; whole-body ^{18}F-FDG PET/CT negative	492 leukocyte/mm^3 (89% mononuclear); hyperproteinorachia (142 mg/dL proteins)	Negative in blood and in CSF.	N/R	Skin culture: negative; negative on serum; positive on CSF for *B. burgdorferi* s.l..	Ceftriaxone IM 2g 1x/d for 21 d/total recovery
(*3*)	France/64 y/F	Follicular lymphoma (complete remission)	Vincristine /cyclophosphamide and Rituximab; 7 y before borreliosis	Tetraparesis, predominantly proximal	Brain MRI negative; spine MRI showed meningeal contrast enhancement; EMG was negative; whole-body ^{18}F-FDG PET/CT was negative	Lympho-mononuclear pleiocytosis; hyperproteinorachia; oligoclonal bands.	Negative in blood, positive in CSF	N/R	N/R	Ceftriaxone for 21 d/total recovery
(*4*)	Netherlands/70 y/M	Mantle cell lymphoma.	R-CHOP, autologous stem cell transplantation, rituximab maintenance; ongoing at the onset of borreliosis	Intermittent fever, myalgia, headaches, tinnitus and hearing loss, exanthema, left facial palsy, unsteady gait, dysphagia, 6kg loss.	Brain MRI showed periventricular white matter lesions, pathological enhancement of multiple cranial nerves; whole-body CT scan was negative; whole-body ^{18}FDG PET/CT was negative	279 leukocyte/mm3 (60% lymphocytes); 255 mg/dL, proteins.	Negative in blood and in CSF initially. IgG p41 weakly positive in blood	N/R	CSF negative for *B. myamotoi*, weakly positive for *B. burgdorferi* s.l. on 2nd sample (negative on 1st sample)	Ceftriaxone IV 2g 1x/d for 21 d/kept mild dysphagia and dysarthria

Study	Country/age/sex	Previous hemopathy	Previous treatment; date of last rituximab treatment	Symptoms	Additional tests	CSF data	*Borrelia* serology	CXCL13	*Borrelia* PCR	Treatment/ outcome
Current study, 2024	Belgium/58 y/F	Follicular lymphoma	R-CHOP and rituximab maintenance; rituximab was ongoing at the onset of borreliosis	Progressively worsening sensory-motor deficit in the distal right lower limb, intense neuropathic pain in the same area and hyperesthesia	Positive GM1 antibodies at a low titer; EMG: axonal lumbar plexus involvement of the right lower limb; brain MRI negative; spine MRI showed subtle intradural contrast enhancement; plexus MRI showed infiltration of the right adductor muscle compartment; Whole-body ^{18}FDG PET negative	33 leukocyte/mm^3 (30% neutrophils, 45% lymphocytes, 25% macrophages); hyperproteinorachia (84 mg/dLproteins); several oligoclonal bands but none were specific to the CSF (“mirror pattern”)	Negative in blood and in CSF	Highly positive (>350pg/ mL)	Positive in CSF for *B. spielmanii*	Ceftriaxone IV 2g 1x/d for 21 d/total recovery

*18F-FDG, ^{18}F-fluorodeoxyglucose; CSF, cerebrospinal fluid; CT, computed tomography; EMG, electromyography; MRI, magnetic resonance imaging; N/R, not reported; PET, positron emission tomography; R-CHOP, rituximab, cyclophosphamide, hydroxydaunorubicin, vincristine, and prednisolone.

References

1. Harrer T, Geissdörfer W, Schoerner C, Lang E, Helm G. Seronegative Lyme neuroborreliosis in a patient on treatment for chronic lymphatic leukemia. Infection. 2007;35:110–3. PubMed https://doi.org/10.1007/s15010-007-6121-0

2. van Dop WA, Kersten MJ, de Wever B, Hovius JW. Seronegative Lyme neuroborreliosis in a patient using rituximab. BMJ Case Rep. 2013;2013:bcr2012007627. PubMed https://doi.org/10.1136/bcr-2012-007627

3. Gampourou F, Taithe F, Moisset X, Clavelou P. Seronegative Lyme neuroborreliosis in a patient treated by rituximab. Rev Neurol (Paris). 2016;172:166–7. PubMed https://doi.org/10.1016/j.neurol.2015.06.009

4. Wagemakers A, Visser MC, de Wever B, Hovius JW, van de Donk NWCJ, Hendriks EJ, et al. Case report: persistently seronegative neuroborreliosis in an immunocompromised patient. BMC Infect Dis. 2018;18:362. PubMed https://doi.org/10.1186/s12879-018-3273-8
